# Molecular mechanism and health effects of 1,2-Naphtoquinone

**DOI:** 10.17179/excli2020-1210

**Published:** 2020-06-03

**Authors:** Antonio G. Soares, Marcelo N. Muscara, Soraia K. P. Costa

**Affiliations:** 1 Department of Cellular and Integrative Physiology, University of Texas Health Science Center at San Antonio, USA. 7703 Floyd Curl Dr. San Antonio, TX, USA 78229; 2 Laboratory of Biochemical Pharmacology of Free Radicals, Inflammation and Pain, Departamento de Farmacologia, Instituto de Ciencias Biomedicas (ICB), University of Sao Paulo, Brazil. Av. Prof Lineu Prestes, 1524 Cidade Universitaria, Sao Paulo, SP CEP 05508-000, Brazil

**Keywords:** naphthoquinone, free radicals, inflammation, cardiovascular system

## Abstract

Extensive literature regarding the health side effects of ambient pollutants (AP) are available, such as diesel exhaust particles (DEPs), but limited studies are available on their electrophilic contaminant 1,2-Naphthoquinone (1,2-NQ), enzymatically derived from naphthalene. This review summarizes relevant toxicologic and biological properties of 1,2-NQ as an environmental pollutant or to a lesser degree as a backbone in drug development to treat infectious diseases. It presents evidence of 1,2-NQ-mediated genotoxicity, neurogenic inflammation, and cytotoxicity due to several mechanistic properties, including the production of reactive oxygen species (ROS), that promote cell damage, carcinogenesis, and cell death. Many signal transduction pathways act as a vulnerable target for 1,2-NQ, including kappaB kinase b (IKKbeta) and protein tyrosine phosphatase 1B (PTP1B). Antioxidant molecules act in defense against ROS/RNS-mediated 1,2-NQ responses to injury. Nonetheless, its inhibitory effects at PTP1B, altering the insulin signaling pathway, represents a new therapeutic target to treat diabetes type 2. Questions exist whether exposure to 1,2-NQ may promote arylation of the Keap1 factor, a negative regulator of Nrf2, as well as acting on the sepiapterin reductase activity, an NADPH-dependent enzyme which catalyzes the formation of critical cofactors in aromatic amino acid metabolism and nitric oxide biosynthesis. Exposure to 1,2-NQ is linked to neurologic, behavioral, and developmental disturbances as well as increased susceptibility to asthma. Limited new knowledge exists on molecular modeling of quinones molecules as antitumoral and anti-microorganism agents. Altogether, these studies suggest that 1,2-NQ and its intermediate compounds can initiate a number of pathological pathways as AP in living organisms but it can be used to better understand molecular pathways.

## Introduction

The actions of 1,2-Naphthoquinone (1,2-NQ) were described in the 1960s when the carcinogenic effects of naphthalene in the bladder were studied and specific markers were sought. The hypothesis was raised by the fact that naphthalene, being enzymatically converted in the liver, could generate some intermediary compound that would be responsible for the development of cancer. In an article published by Ollodart and Rose a description of anti-1,2-NQ antibodies to detect plasma markers of these naphthalene intermediate compounds demonstrating that this compound plays a role in the organism (Ollodart and Rose, 1962[38]).

A few years later, an important study with *C. elegans* demonstrated that the metabolism of naphthalene confirmed the production of intermediate metabolites: 1-naphthol (67.9 %) and 4-hydroxy-1-tetralone (16.7 %). Other minor products isolated were 1,4-naphthoquinone (2.8 %), 1,2-naphthoquinone (0.2 %), 2-naphthol (6.3 %), and trans-1,2-dihydroxy-1,2-dihydronaphthalene (5.3 %) thus confirming the conversion of naphthalene to various other intermediate compounds with potential toxicity (Cerniglia and Gibson, 1977[7]).

In 2000, Bolton and colleagues[5] published an important review article summarizing the toxic effects of quinones in the body. The article presents the main characteristics that quinones can promote their harmful effects in living organism. Quinones are acceptors in Michael's reaction, they can promote cell damage through the alkylation of cellular proteins and DNA. Also, they are highly active molecules with semiquinone radicals' formation, leading to production of reactive oxygen species (ROS), including superoxide (O_2_^-^), hydrogen peroxide (H_2_O_2_) and hydroxyl radical (OH). Additionally, ROS production can cause severe oxidative stress within cells through the formation of oxidized cell macromolecules, including lipids, proteins, and DNA. DNA bases damaged by oxidation, such as 8-oxodeoxyguanosine, has been associated with aging and carcinogenesis as a result of quinone exposure. Besides, ROS can also activate several signaling pathways, including protein kinase C and RAS contributing even more to cell damage (Bolton et al, 2000[5]).

## Toxic Effects of 1,2-Naphthoquinone

The toxic effects promoted by 1,2-NQ were studied in several cellular lines. For example, using hepatocytes treated with 1-naphthol, 1,2-, and 1,4-NQ in increasing concentrations showed a reduction in GSH levels, culminating with cell death (cytotoxicity) (Doherty et al., 1984[13]). The search for therapies for the treatment of melanomas led to a study which observed that formation of 1-2-NQ was due to a reaction promoted by the tyrosinase (produced in epithelial cells) upon 1-naphthol, suggesting the possible cytotoxic effect of 1,2-NQ because of 1-naphthol exposure (Doherty et al., 1985[12]). Interestingly, in a study published by Miller et al. (1986[32]), in which the possible conjugation of 1-naphthol and naphthoquinones (1,2- and 1,4-) was studied, GSH conjugates were not detected when the samples were incubated with 1,2-NQ. The authors suggest that they may have failed to detect naphthoquinones because of the high reactivity of these compounds (Miller et al., 1986[32]).

Studies of cytotoxicity and genotoxicity were performed by Wilson et al. (1996[61]) and it was observed that both 1,2-NQ and 1,4-NQ are toxic at concentrations higher than 50 micro molar in whole blood samples after chromosome analysis. Also, it was observed that concentrations greater than 0.1 µM promoted an increase in the cell death rate (Wilson et al., 1996[61]).

In an article published by Shang et al. (2014[43]), the potential protective effects of N-acetylcysteine were evaluated in an *in vitro* model of quinone exposure. The authors observed that 1,2-NQ promoted its cytotoxic and genotoxic effects, but the treatment was effective in reversing such 1,2-NQ actions (Shang et al., 2014[43]). Sheng and Lu (2017[44]) demonstrated that 1,2-NQ leads to increased expression of several pro-inflammatory markers in human lung cells (A549): IL-6, IL-8, TNF-α, IL-8, TNF-α, IL-8, Cyp1a1, and hemeoxygenase-1 (HO-1) (Sheng and Lu, 2017[44]). In another study. Abiko et al. (2016[1]) demonstrated the activation of the AhR (Aryl) receptor by the quinones in HepG2 cells (liver tumor cell lines) and the translocation of this receptor to the nucleus (Abiko et al., 2016[1]). Nishina et al. (2017[37]) demonstrated that 1,2-NQ leads to IL-11 production via ERK activation, and a nuclear interaction of two AP-1 promoter sites (Nishina et al., 2017[37]). Finally, Lavrich et al. (2018[26]) have demonstrated that H_2_O_2_ production by 1,2-NQ occurs in mitochondrial human lung epithelial cells (Lavrich et al., 2018[26]).

All these data demonstrated that 1,2-NQ and its intermediate compounds could trigger a number of inflammatory pathways in different cells, suggesting that the effects of this compound in the organism are harmful due to this great range of action in different cells and tissues.

## 1,2-Naphthoquinone Mechanisms of Action

This topic of the review will focus on the different targets of 1,2-NQ, the description of several targets shows that the impact of exposure to such contaminants could disrupt the insulin pathway, lead to ROS formation and inflammation. Also, 1,2-NQ could be used as a tool to get a better understanding of several molecular pathways involved in different pathological conditions.

The inhibitory effect of 1,2-NQ on the catechol-1,2-oxygenase enzyme (Varga and Neujahr, 1972[57]) was described in 1972. Another target was described by Nesnow et al. (1980[36]) on benzopyrene oxidation and the interaction of 1,2-NQ with NADPH. The authors demonstrated that the electrophilic potential of 1,2-NQ was confirmed by 1,2-NQ reactions with NADPH, and that 1,2-NQ interactions could occur in oxygen dependent hepatic enzyme systems (Nesnow et al., 1980[36]).

Superoxide anion formation was initially demonstrated in the hepatic microsomal system in an article published by Thornalley et al. (1984[54]). The toxicity study of 1-naphthol and naphthoquinones (1,2- and 1,4-NQ) showed the production of superoxide anion in the presence of NAD and NADPH in hepatocytes of rats. Furthermore, this reactive oxygen species production system was abolished when the cells were treated with superoxide dismutase (SOD). This work, sought to understand the mechanisms by which 1-naphthol exerts its toxic effect on tumor cells, allowing a better understanding of the toxic actions of naphthoquinones (Thornalley et al., 1984[54]).

In a study published by Zheng et al. (1997[63]) it was shown that 1,2-NQ can react with cysteine residues through covalent bonds, thereby altering the protein structure (Zheng et al., 1997[63]). An important study by Ahn et al. (2002[3]) shows that 1,2-NQ inhibits PTP1B (protein tyrosine phosphatase 1B), a major negative regulator of insulin signaling, which plays an important role in the development of diabetes type 2 (Ahn et al., 2002[3]; Kennedy and Ramachandran, 2000[21]). In this study, the authors sought to create new molecules that presented similar characteristics to the structure of 1,2-NQ, since in the condition of type 2 diabetes, inhibition of PTP1B could be a new therapeutic target for the treatment of this disease (Ahn et al., 2002[3]; Cheon et al., 2004[9]).

Troester and colleagues sought to determine potential biomarkers to assess the toxic capacity of naphthalene and quinone exposure. However, as shown previously, the adducts of the reaction between 1,2-NQ and serum proteins showed to be very unstable and difficult to detect (Troester et al., 2002[56]). Waidyanatha et al. (2002[59]) again detected changes in serum proteins (hemoglobin and albumin) by gas chromatography-mass spectrometry after cleavage and derivatization of the adducts with trifluoroacetic anhydride and methane sulfonic acid. In this study, it was observed that after exposure of mice to naphthalene at a dose of 100 to 800 mg/kg, the production of 1,2-NQ adducts in hemoglobin and albumin could be detected in a dose-dependent manner (Waidyanatha et al., 2002[59]).

Another important study published by Waidyanatha et al. (2004[60]) carried out translational studies to verify the existence of biomarkers for exposure to naphthalene and polycyclic hydrocarbons in individuals working in steel industries in China. The study presented interesting results, since it was able to detect albumin adducts for both 1,2-NQ and 1,4-NQ. Detection levels, on the picomol range, were effective on individuals working in furnaces. In addition, it was possible to correlate the exposure time with serum levels of albumin adducts (1,2-NQ-Alb) (Waidyanatha et al., 2004[60]). Lin et al. (2009[28]) studied the cumulative effects of exposure to naphthoquinones in healthy individuals in Taiwan, and the authors observed that median concentrations of the 1,2-NQ adduct in humans were 268 pmol/g (range 139-857 pmol/g; n=11) in males, and 203 pmol/g (range 128-1352 pmol/g; n=11) in women.

Kikuno and colleagues demonstrated that exposure of guinea pig tracheas to 1,2-NQ can, through covalent binding, cause phosphorylation of protein tyrosine (PTP) kinase, leading to the activation of the pathway phospholipase A2/lipoxygenase/TRPV1. Furthermore, in the same work the authors showed that 1,2-NQ can also promote protein changes through covalent binding to thiol groups (Kikuno et al., 2006[22]). Also, in another study the inhibition of PTP was associated with increased EGFR activation in lineage epithelial cells (Iwamoto et al., 2007[18]).

Interesting data shown by Sun and colleagues (2006[51]) on the effects of 1,2-NQ on rabbit aorta demonstrated that increasing concentrations of 1,2-NQ (1 to 5 µM) have the ability to significantly reduce vasodilation and eNOS enzyme activity. The authors conclude that the effects of 1,2-NQ on eNOS occurs in a manner dependent on the redox state produced by the compound itself by an interaction with the eNOS structure (e.g. via covalent change of thiol groups; (Sun et al., 2006[51])).

Endo and colleagues (2007[16]) presented another mechanism by which 1,2-NQ can promote its toxic effects. In this study, it was possible to observe that CREB (cyclic AMP responsive binding protein), a transcription factor with cysteine residues, and target for covalent binding of 1,2-NQ to thiol moieties, had reduced nuclear DNA binding activity (Endo et al., 2007[16]), and that this inhibition was due to the structural modification at the Cys-286 (Endo et al., 2011[15]).

Other inhibitory effects from the interaction with 1,2-NQ were demonstrated by Shimada et al. (2007[45]). Extracts from different tissues (liver, kidneys and lungs) were prepared and incubated with 1,2-NQ (10 µM). 20α-HSD which catalyzes the reduction of progesterone to its inactive metabolite 20α-hydroxy-4-pregnen-3-one, and it is involved in the regulation of the amount of progesterone that binds to its nuclear receptor, had its activity reduced to 50% in the presence of 1,2-NQ, while in lung and kidney samples the inhibition of the same enzyme was approximately 10% (Shimada et al., 2007[45]).

In a previous study by our group, animals treated intrathecally with a mixture of DEP and 1,2-NQ (35-100 nmol) promoted increased mRNA expression of TRPV1, NK1 and NK2 in rat bronchi, partially reversed by pretreatment with capsaicin (Costa et al., 2010[11]).

Sumi and colleagues (2010[50]) verified the effects of 1,2-NQ on the inducible nitric oxide synthase (iNOS) isoform in RAW264.7 cells treated with LPS. The authors observed that treatment with 1,2-NQ was able to reduce NO levels via iNOS, possibly by decoupling the enzyme. They also observed that in lungs from mice treated with LPS, this effect was reproduced, and in addition, they verified that the exposure to 1,2-NQ also promoted alteration in the signaling pathway of IKKbeta/NF-κB, by inhibiting the translocation of the p65 fraction (Sumi et al., 2010[50]).

In a study published by Miura et al., 1,2-NQ promoted arylation of the Keap1 factor, a negative regulator of Nrf2. The exposure of primary rat hepatocytes to 1,2-NQ resulted in activation of Nrf2 and upregulation of some of the Nrf2-activated genes. The authors also demonstrated that NADPH: quinone oxidoreductase 1 and uridine 5'-diphosphate-glucuronosyl transferases are responsible for the detoxification process of hepatocytes exposed to 1,2-NQ, and that Keap1/Nrf2 pathway is important for the redox defense when exposed to this environmental contaminant (Miura et al., 2011[35]).

Also, in another study, Miura and colleagues demonstrated that glyceraldehyde-3-phosphate dehydrogenase (GAPDH) could be another target for structural modification. Through *in vitro* assays the authors demonstrated that A549 lineage human epithelial cells underwent a minor structural change in GAPDH, and the treatment of cells with increasing concentrations of GSH was able to reverse the effect of 1,2-NQ on the formation of adducts of GAPDH-1,2-NQ (Miura et al., 2011[33][34]).

Takayama and colleagues (2011[52]) observed that in A549 lung epithelial cells, the exposition to 1,2-NQ leads to arylation of peroxiredoxin 6, a protein with activities of glutathione peroxidase and phospholipase A2 and of great importance in the antioxidant defense, through the formation of covalent bonds at several sites (Cys47, Lys144 and Cys91), thus resulting in reduction of phospholipase A2 activity (Takayama et al., 2011[52]). Another study focusing on the identification of new targets for 1,2-NQ published by Gurbani and colleagues demonstrated, by binding assays, the potential of 1,2-NQ for blocking the enzyme topoisomerase-II (Gurbani et al., 2012[17]).

Shinkai and colleagues (2012[47]) demonstrated that RAW264.7 cells exposed to 1,2-NQ had a reduction in the activity of thioredoxin 1 (Trx1) due to covalent bonds at the Cys32 and Cys35 residues of the enzyme, thus increasing the list of enzymes and proteins subject to the actions of 1,2-NQ (Shinkai et al., 2012[47]).

New studies have demonstrated the importance of more sophisticated techniques to detect the adducts formed in the organism when exposed to 1,2-NQ. Pham and collaborators (2012[40]) using high resolution mass spectrometry were able to identify binding sites, relative rates of unchanged peptide depletion and binding selectivity to amino acid residues. By this method it can be concluded that the formation of adducts occurred in the cysteine, lysine and histidine residues and in the N-terminal chain of several proteins (Pham et al., 2012[40]).

Another target described is sepiapterin reductase, an NADPH-dependent enzyme that catalyzes the formation of dihydrobiopterin (BH2), a precursor of tetrahydrobiopterin (BH4), a critical cofactor in aromatic amino acid metabolism and nitric oxide biosynthesis. Using recombinant human enzyme, sepiapterin reductase has been shown to be highly efficient in mediating the chemical redox cycle and ROS generation. Interestingly, the redox cycle markedly reduced the ability of the enzyme to generate BH2. Unlike other enzymes that mediate the redox cycle, sepiapterin reductase does not depend on flavins or other cofactors, thus suggesting that it works by a single mechanism. These results are interesting as they identify a flavin-independent pathway mediating the chemical redox cycle in lung epithelial cells. In addition, they demonstrate that redox chemicals can control a key enzyme necessary for the biosynthesis of a major cofactor in the generation of mediators that regulate lung function. The authors observed that quinones react with sepiapterin reductase inhibiting the enzyme's ability to reduce sepiapterin, being 1,2-NQ one of the most potent to block sepiapterin reduction (Yang et al., 2013[62]).

Beei and colleagues (2013[4]) observed that increased EGRF phosphorylation occurred in lung epithelial cells (A459) when exposed to 1,2-NQ, with the consequent activation of the intracellular signaling pathway MEK/ ERK, which leads to the activation of the transcription factor AP-1 (Beei et al., 2013[4]).

Lin and collaborators (2014[27]) demonstrated that quinones (1,2- and 1,4-NQ) can, in addition to form serum-albumin adducts, also react with estrogen to form adducts, which were detected in plasma and correlated with increased risk for development of breast cancer in women (Lin et al., 2014[27]).

In another study by our group, Santos et al. (2014[42]) observed that postnatal exposure to 1,2-NQ increased susceptibility to ovalbumin-induced asthma in mice. The mechanism seems to be dependent on increased expression of co-stimulatory molecules, which leads to amplification of the cellular presentation, enhanced Th2 and LTB4 polarization, humoral response, and Th1/Th2 cytokines (Santos et al., 2014[42]).

Toyama and colleagues (2014[55]) demonstrated another site where 1,2-NQ could promote covalent attachment. In neuroblastoma cells, the authors observed that 1,2-NQ, via GSH, promoted transarylation at the catalytic site of ubiquitin carboxyl-terminal hydrolase L1 (UCH-L1), in the Cys152 portions, leading to inhibition of the enzyme (Toyama et al., 2014[55]).

Shinkai and collaborators (2015[46]) sought to understand the role of thiol groups in the formation of 1,2-NQ adducts, and observed that the process of protein adduction by 1,2-NQ through a thioether bond (CSC) slowly decreases through of a GSH-dependent S-transarylation reaction, whereas the formation of 1,2-dihydroxynaphthalene-4-thiol (1,2-NQH2-SAc) occurred through a disulfide bond (CSSC), which was rapidly restored to the protein free in cells. This latter component, in turn, was able to activate Nrf2, leading to increased expression of genes related to antioxidant defenses. This work demonstrated another chemical reaction that can occur with 1,2-NQ by the sulfidation process (Shinkai et al., 2015[46]).

Abiko and colleagues (2015[2]) demonstrated that 1,2-NQ can activate the cytochrome P450 CYP1A1 in C35 cells via the aryl hydrocarbon receptor (AhR) and further promoted the expression of antioxidant defense-related genes (Abiko et al., 2015[2]). Wages and co-workers (2015[58]), in turn, demonstrated the effects of 1,2-NQ on BEAS-2B cells at increasing concentrations and assessed the potential sulfonylation of GAPDH and PTP1B proteins (Wages et al., 2015[58]).

Finally, Carratt et al. (2016[6]) demonstrated the effects of naphthalene and its derivatives (1,2-NQ) on male and female mice knocked-out for the microsomal enzyme epoxy hydrolase (ehM), responsible for the conversion of naphthalene to its derivatives in the lung. They observed that exposure to naphthalene promoted lung injury only in naive animals, with significant differences between males and females, thus excluding the role of ehM in the deleterious effects promoted by naphthalene and its derivatives on the lungs of exposed animals (Carratt et al., 2016[6]).

The large amount of targets involved in 1,2-NQ exposure shows how complicated is to understand the different mechanisms involved in the different molecular pathways, demonstrating, once more, the importance to understand the toxic effects of quinones.

## 1,2-Naphthoquinone as Backbone Molecule for the Development of New Drugs

By 1970, the toxic effects of naphthalene were already known, but the mechanisms by which this compound promoted its toxic actions in the body were not fully understood. Another interesting aspect of quinones, particularly 1,2-NQ, is that several studies describe the effects of 1,2-NQ as precursor of new drugs for the treatment of cancer, as well as antiparasitic and antifungal.

In 1978, Lopes and colleagues studied the toxic effects of 1,2- and 1,4-NQ for treatment against *T. cruzi*, but these compounds were not effective in reducing the infectivity of *T. cruzi* when exposed to different concentrations of these quinones. Much was observed since naphthoquinones can react with serum proteins and thus lose the effect on the microorganisms studied (Lopes et al., 1978[30]).

Interestingly, some drugs, such as menandione, when associated with 1,2-NQ have antitumor actions which were addressed in several studies. Furthermore, another lapachone structure associated with the 1,2-NQ structure received great attention, since these structures have the potential to promote the release of ROS and thus have shown to be promising, not only for the treatment of some tumor types, but also as effective antiparasitic and antifungal agent (Meazza et al., 2003[31]; Dolan et al., 1998[14]; Shukla et al., 2012[48]; Lin et al., 2005[29]). Several studies have sought inhibitors based on the structure of 1,2-NQ through molecular modeling (Q-SAR) (Pahwa and Papreja, 2012[39]).

## Induction of Cataract by Exposure to 1,2-Naphthoquinone

Another toxic characteristic studied of 1,2-NQ is its potential to promote cataracts. In a study by Kleber et al. (1991[23]), 1,2-NQ-exposed bovine retinas developed cataracts due to depletion of thiols, NADH and ascorbate in the aqueous portion of the eye (Kleber et al., 1991[23]), as well as the formation of hydrogen peroxide (Kröner et al., 1991[24]).

Qian and Shichi (2001[41]) observed that after the intraocular injection of 1,2-NQ, there was an increase in Ca^2+^ ion concentrations and calpain activation. These results suggest that 1,2-NQ is most likely promoting calcium channel alterations, although the study did not even propose which of these channels would be involved (Qian and Shichi, 2001[41]).

Finally, Jacob and colleagues evaluated the effects of 1,2-NQ on membrane lipid oxidation and observed that exposure to 1,2-NQ led to the formation of lipoperoxides (LOOH), thus suggesting a mechanism by which 1,2-NQ may be present promoting the toxic effects on cataract induction by exposure to naphthalene as previously described (Jacob et al., 2013[19]).

## 1,2-Naphthoquinone as an Environmental Pollutant

Diesel fumes produced by vehicle motors release in the air tons of contaminants every day, and among these contaminants, 1,2-NQ plays an important role in the development of several pathological conditions, as mentioned above.

Kumagai and colleagues (1995[25]) demonstrated the effects of 1,2-NQ as a contaminant of the exhaust particles from diesel combustion, to block SOD and to react with superoxide generating hydroquinone and a semiquinone radical capable of reducing cytochrome c. In addition, the authors suggest that the quinones present in the particles from the diesel exhaust could serve as a substrate for NADPH-P450 reductase, thus maintaining high levels of superoxide production and demonstrating the toxic potential of DEP and its contaminants (Kumagai et al., 1995[25]). Additionally Terada and collaborators (1995[53]) observed the inactivation of glutathione S-transferase subtype π after incubation with 1,2-NQ (Terada et al., 1995[53]).

Two studies sought to determine the concentration of contaminants present in the gases and particulate matter emitted in the combustion process of diesel and gasoline. In the study by Cho and co-workers, they were able to determine the 1,2-NQ with a limit measurement of up to 300 pg (Cho et al., 2014[10]), so the concentration of 13.7 µg/g DEP was calculated. Jakober et al. (2007[20]) were able to estimate the concentration of 1,2-NQ ranging from 10 to 340 µg/L in tests performed with different types of engines and detection methods that evaluated fuel types (gasoline and diesel) (Jakober et al., 2007[20]).

Once established that 1,2-NQ may lead to cellular damage, several studies sought to understand the effects related to exposure to this pollutant. Cheng et al. (2012[8]) evaluated the exposure of pulmonary epithelial cells (BEAS) to understand the effects of 1,2-NQ on the inflammatory and gene response. The authors observed increased production of hydrogen peroxide, leading to increased expression of inflammatory genes such as IL-8 and COX-2. Treatment of cells with catalase reversed these effects. In addition, the authors also demonstrated that mitochondria were the source of H_2_O_2_ production. Much of that observed in this study has been correlated with previous literature data where DEP and other contaminants may promote effects like those seen with 1,2-NQ (Cheng et al., 2012[8]).

## Effects of 1,2-Naphthoquinone on the Cardiovascular System

The effects of 1,2-NQ as observed above could trigger a great number of molecular pathways involved in the development of several pathological conditions. Among these, arrhythmias and hypertension would be expected. For example, our group demonstrated that rats exposed to 1,2-NQ showed increased vasorelaxation capacity in the aorta and cavernous body of rats (unpublished data). Furthermore, exposure of male mice to 1,2-NQ during the neonatal phase led to endothelial dysfunction in the pulmonary artery of these animals. In another work, we demonstrated that 1,2-NQ can promote positive chronotropism in the right atrium of mice, possibly via β1 adrenergic receptors and their interaction with TRPV1 receptors (Soares et al., 2016[49]). 1,2-NQ promotes vasoconstriction in the pulmonary and mesenteric arteries isolated from mice, and blockade of TRPA1 and TRPV1 demonstrated specific responses to each vascular bed studied (Soares et al., 2016[49]).

## Conclusion

In conclusion, the long list of targets that 1,2-NQ may interact with shows how difficult it is to get a full picture of just one compound in the organism. It is well known that naphthalene and its intermediaries (including 1,2-NQ) show toxic effects to several organisms, however, the mechanisms involved are still to be fully understood.

## Conflict of interest

The authors declare that they have no conflict of interest.
